# Unveiling MHC-*DAB* Polymorphism Within the Western Balkan Salmonid Hotspot: Preliminary Outcomes from Native Trouts of Ohrid Lake and the Drin-Skadar Drainage (Albania)

**DOI:** 10.3390/biology13121060

**Published:** 2024-12-18

**Authors:** Lorenzo Talarico, Arnold Rakaj, Lorenzo Tancioni

**Affiliations:** 1 Laboratory of Experimental Ecology and Aquaculture, Department of Biology, University of Rome “Tor Vergata”, Via Cracovia 1, 00133 Rome, Italytancioni@uniroma2.it (L.T.); 2 National Inter-University Consortium for Marine Sciences (CoNISMa), Piazzale Flaminio 9, 00196 Rome, Italy

**Keywords:** *Salmo letnica*, *Salmo ohridanus*, *Salmo trutta* complex, major histocompatibility complex, positive selection, evolution

## Abstract

Due to their involvement in pathogen-mediated immune responses, the hypervariable genes of the Major Histocompatibility Complex (MHC) have become a paradigm for investigating the evolution and maintenance of genetic (adaptive) diversity, contextually providing insight into the viability of wild populations, which is meaningful for conservation. Here, we provide the first preliminary characterization of MHC polymorphism and evolution in trouts from Albania, a known hotspot of Salmonid diversity harboring ecologically and phylogenetically distinct native (threatened) taxa. Overall, 36 trout—including Lake Ohrid-endemic *Salmo ohridanus* and *S. letnica*, and both riverine and lacustrine native brown trout (the *S. trutta* complex) from the Drin-Skadar drainage—were genotyped at the MHC-*DAB* locus through next-generation amplicon sequencing. We identified 34 alleles (including 30 novel alleles), unveiling remarkable population/taxon MHC-*DAB* distinctiveness. Despite apparent functional (supertype) similarity, *S. letnica* and the *S. trutta* complex showed MHC-typical high sequence/allele diversity and evidence of global/codon-specific positive selection, particularly at antigen-binding sites. Conversely, deep-water-adapted *S. ohridanus* revealed unexpectedly reduced allelic/supertype diversity and relaxed selection. Evolution by reticulation and signals of trans-species polymorphism emerged from sequence genealogies. Further investigations and increased sampling will provide a deeper understanding of the evolutionary mechanisms yielding the observed pattern of MHC diversity across Albanian trout taxa and populations.

## Simple Summary

We provide the first (preliminary) characterization of an immune-related gene (MHC-*DAB*) in trouts from Albania, an area harboring ecologically and genetically rich Salmonid diversity, including endemisms valuable for conservation. By genotyping 36 trout samples, we revealed generally high sequence/allelic polymorphism and remarkable distinctiveness (34 different sequences in total, most of which were exclusive to a taxon or population), also finding expected signals of historical positive selection. Interestingly, samples of lacustrine Belvica trout (*Salmo ohridanus*) showed contrasting results compared to other examined trout (*S. trutta* complex and *S. letnica*). Further investigations will provide a deeper understanding of the evolutionary mechanisms yielding the observed pattern of MHC-*DAB* diversity. Beyond evolutionary genetics, our outcomes offer useful information (namely immune-related adaptive genetic diversity) for conservation programs aimed at preserving the long-term viability of threatened wild populations.

## 1. Introduction

Salmonids of the genus *Salmo* are amongst the most polymorphic and studied taxa, showing multiple genetic lineages and morpho-ecological forms that are frequently associated with local distribution. This led to the description of numerous, often conflicting, (sub)species [1], so *Salmo* taxonomy remains unresolved [2]. Despite this taxon originally inhabiting the Palaearctic Region, in the last centuries it has been intensively farmed and introduced almost worldwide, mostly for recreational angling [1].

In Albania, which is part of the Western Balkan *Salmo* (genetic) diversity hotspot, both riverine and lacustrine native brown trout occur, which formerly belonged to the *Salmo trutta* species complex [3]. Among these is the Ohrid trout (referred to as *S. letnica*), a lake-dwelling trout endemic to Ohrid Lake and attributable to the “Adriatic” mitochondrial lineage of *S. trutta* complex [4,5]. Another taxon of remarkable conservation and biological value is *S. ohridanus* (Belvica trout), a lake-resident and morphologically distinctive trout adapted to inhabit the deep waters of Ohrid Lake. It constitutes an ecologically and genetically well-defined taxon that diverged >4 Mya from the *S. trutta* complex, from which it is reproductively isolated–natural hybridization with the sympatric *S. letnica* seems negligible [1,4]. Besides their intrinsic vulnerability as endemisms, native Albanian trout populations are threatened by multiple anthropic pressures, among which the most impactful are habitat fragmentation and degradation, (illegal) fishing, trophic competition, and introgressive hybridization with farmed exotic lineages stocked for fishery enhancement [6].

In jawed vertebrates, classical genes of the Major Histocompatibility Complex (MHC) encode for membrane-bound proteins, each recognizing (and binding) a limited spectrum of parasite/pathogen-derived antigens, eventually presenting them to cells of the immune system and activating the immune response [7]. The antigen-binding specificity of MHC proteins is determined by amino acids at particular positions (i.e., antigen-binding sites (ABSs)) that are mostly scattered across exon 2 in MHC class IIB genes. Because of this, positive selection promotes intense amino acid replacement, especially at ABSs, resulting in MHC-typical high levels of sequence diversity and, in turn, numerous alleles. Such high MHC polymorphism, frequently increased by gene duplication and recombination, should confer resistance against a broader antigen spectrum [8]. Importantly, MHC polymorphism often persists over evolutionary time—likely maintained by mechanisms of pathogen-mediated balancing selection and, possibly, sexual selection [9]—so similar or identical alleles can be found even in deeply divergent taxa, i.e., the trans-species polymorphism phenomenon [8]. Because of its features, the MHC has become a paradigm for investigating pathogen-mediated selection and the evolution of adaptive diversity, contextually providing meaningful insight into the viability of wild populations and a baseline for their conservation [10].

A single classical MHC class IIB gene exists in Salmonids, i.e., MHC-*DAB* [11]. Previous studies investigated the evolution of MHC-*DAB* exon 2 polymorphism in *S. trutta* complex wild riverine populations from central Italy [12], northern Spain [13], and Austria [14], demonstrating recent/historical selection and diversity of various degrees. However, putatively adaptive MHC diversity remains completely unknown in the Western Balkan trout hotspot to date, especially for endemic lacustrine taxa possibly exposed to peculiar pathogen pressures. Here, taking advantage of opportunistic sampling and next-generation amplicon sequencing-based genotyping, we provide a preliminary characterization of the MHC-*DAB* exon 2 polymorphism of *S. letnica* and *S. ohridanus* from Ohrid Lake and both lacustrine and riverine *S. trutta* complex from the Drin-Skadar drainage (Albania).

## 2. Materials and Methods

During the summer of 2019, we obtained 36 wild trout specimens caught by local professional fishermen in Albania: 22 *S. letnica* and 3 *S. ohridanus* from Ohrid Lake, plus 11 *S. trutta* complex specimens from Skadar Lake and its tributary, the Cem River (5 and 6 specimens, respectively), all within the Drin-Skadar drainage (Table 1). Fin clips were stored in ethanol at −20 °C until DNA extraction, which followed the mammalian tissue protocol of the GenElute™ Mammalian Genomic DNA Miniprep Kit (Sigma-Aldrich, St. Louis, MO, USA).

First, to validate the specimens, we PCR-amplified and Sanger-sequenced a fragment of the taxonomically informative mitochondrial Control Region (CR) following procedures detailed in [15].

To assess MHC variation, we PCR-amplified with individually tagged primers a 254–257 bp fragment of exon 2 of the MHC class II *DAB* gene, for which several comparable data were available in the literature for native *Salmo* populations across Europe. Tagged amplicons were pooled in batches and sequenced in a 2 × 300 bp Miseq run (Illumina, San Diego, CA, USA) along with *Salmo* MHC amplicons from another study. Laboratory and bioinformatics procedures, i.e., data processing, quality control, and MHC genotyping through AmpliSAT tools [16], were performed as in [12].

MHC-*DAB* alleles of *S. letnica* and *S. ohridanus* were named according to [17], while novel alleles of the *S. trutta* complex continued the numeration in [12]. We aligned sequences with ClustalW in MEGA11 [18] and reconstructed relationships through a neighbor-net network (Kimura two-parameter distance) in SplitsTree5 since this method is effective in cases of intricate phylogeneses [19]. We explored possible recombination breakpoints in the alignment using the GARD algorithm [20] implemented in Datamonkey (http://www.datamonkey.org/; accessed on 10 April 2024).

For each taxon separately, we estimated MHC-*DAB* sequence polymorphism as the mean pairwise nucleotide distance (the Kimura two-parameter model correcting for multiple hits); the mean pairwise amino acid distance (the Poisson correction model); and the rates of synonymous (dS) and non-synonymous (dN) substitutions (the Nei–Gojobori method with the Jukes–Cantor correction). The measures were computed in MEGA11 including all sites, 23 sites corresponding to human MHC antigen-binding sites (ABSs) [21], and non-ABSs.

We inspected signatures of historical positive selection in MHC sequences for each taxon separately via a one-tailed Z-test for an excess of dN over dS mutations (the Nei–Gojobori method with the Jukes–Cantor correction and 1000 bootstrap replicates) in MEGA11. Sites under positive selection (PSSs) were inferred by four methods implemented in Datamonkey – fixed-effects likelihood (FEL), fast unconstrained Bayesian approximation (FUBAR), tmixed-effects model of evolution (MEME), and single-likelihood ancestor counting (SLAC) [22,23,24], conservatively considering only sites detected by at least two methods.

We determined theoretical supertypes, namely groups of functionally similar alleles, based on the physicochemical properties at sites involved in antigen binding. To do so, we followed the procedure in [25]: alleles were characterized by five z-descriptors at each PSS, then clustered according to the K-means algorithm implemented in the Adegenet R package [26]. The optimal number of supertypes coincided with the last increase in the cluster number reducing BIC by > 2 (i.e., the ΔBIC > 2 criterion).

To assess and compare MHC-*DAB* variation among populations, we used traditional indices of population diversity: the number of alleles (A) and the observed (Ho) and expected (He) heterozygosities (also testing for deviations from Hardy–Weinberg expectations) were calculated in GenAlEx 6.5 [27]. The PopGenReport 3.1 R package [28] was used to calculate allelic richness by rarefaction (minimum number of sampled alleles = 6 in *S. ohridanus*) to account for unequal sample sizes. Finally, Fst and Jost’s D (which is appropriate in cases of multi-allelic, highly heterozygous markers [29]) estimates of MHC-based population/taxa differentiation were computed in GenAlEx 6.5, and their significance was evaluated through 999 permutations.

## 3. Results

**Figure 1:**
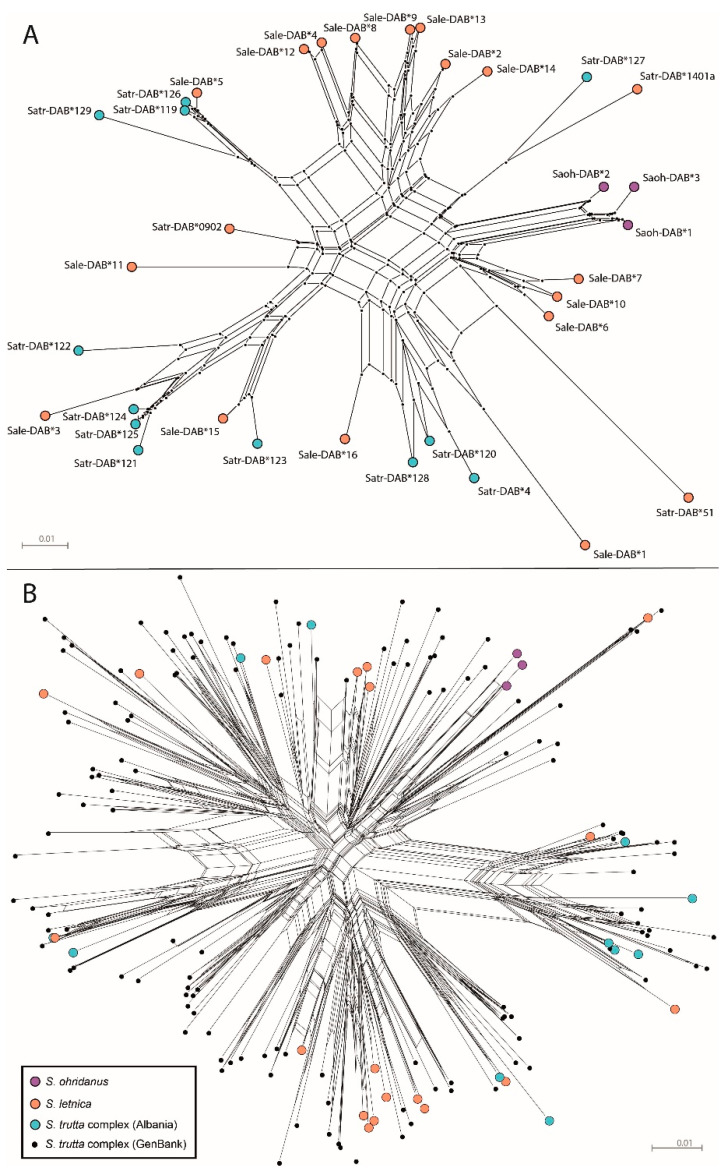
Neighbor-net network (Kimura two-parameter nucleotide distance) depicting relationships among MHC-*DAB* exon 2 sequences: (**A**) 36 *Salmo* taxa alleles from this study and (**B**) 169 *S. trutta* complex alleles retrieved from GenBank. For better visualization, labels indicating codes of MHC-*DAB* alleles are not shown in Figure 1B.

Sequencing of the CR resulted in fragments of 555 bp, all matching with sequences deposited in GenBank, eventually confirming the taxonomic attribution for each specimen (for details see Table S1). Processed Illumina raw data returned on average 4174.5 (±2062.5 SE) reads per MHC amplicon, 65.6% (±21.2% SE) of which corresponded to actual allele reads after AmpliSAS genotyping. Individuals showed 1–2 alleles each. Overall, we obtained 34 MHC-*DAB* alleles of 254 or 257 bp, translating into 31 unique sequences of 85 amino acids with no stop codons (Figure S1). Novel MHC-*DAB* alleles represented 84.2% of alleles in *S. letnica* (16 out of 19 alleles; the known alleles were MHC-*DAB* * 051, * 0902, and * 1401a), 91.7% in *S. trutta* complex (11 out of 12; the known allele was MHC-*DAB* * 004), and 100% in *S. ohridanus* specimens (3 alleles) (Figure S1 and Figure 1A).

The GARD analysis revealed a single putative recombination breakpoint at nucleotide position 174 (Figure S1). The neighbor-net network depicting the MHC-*DAB* allele genealogies showed generally high dissimilarity between sequences whose divergence degree appeared unrelated to taxon membership, although *S. ohridanus* alleles clustered together (Figure 1).

Concerning sequence polymorphism, irrespective of taxa, both nucleotide and amino acid sequence diversity increased (up to five-fold) from the non-ABS partition to the ABS partition, with intermediate values when considering all sites (Table 2). Similarly, both the dN and dS substitution rates were lower at non-ABSs and higher at ABSs across taxa. However, dN was statistically higher than dS (*p* < 0.01) in all taxa except *S. ohridanus* when tested within the ABS partition or including all sites (Z tests; Table 2). The *S. ohridanus* sequences showed lower polymorphism.

Tests for positive selection at the codon level detected from 4 (SLAC) to 15 (FUBAR) putative PSSs, 10 of which were identified by at least two methods, namely amino acid positions 7, 9, 13, 21, 53, 68, 75, 78, 81, and 83 (Figure S1). Based on the physicochemical properties of the 10 assumed PSSs, the MHC-*DAB* alleles were clustered into four supertypes: ST1 = 12 alleles, ST2 = 8 alleles, ST3 = 8 alleles, and ST4 = 6 alleles (Figure S1). Except for three *S. ohridanus* alleles that fell within ST4, the MHC-*DAB* alleles of *S. letnica* and *S. trutta* complex were scattered across all supertypes (Figure 1 and Table 1).

The MHC-*DAB* diversity across populations is summarized in Table 1. The maximum and minimum allelic diversities (A and Ar) were found in *S. letnica* and *S. ohridanus,* respectively, while *S. trutta* complex populations of Cem and Skadar revealed similar intermediate diversities. Both Ho and He were generally high in all populations except in Cem, which showed a significant Ho deficiency. Allele/supertype frequencies and spatial distributions are shown in Figure S2. Population differentiation was generally high overall (Fst = 0.19 and D = 0.99, *p* < 0.002) and between most pairs (Table 3).

## 4. Discussion

Here, we provide the first characterization of MHC-*DAB* polymorphism and population diversity in a reduced sample of native/endemic *Salmo* taxa with remarkable conservation value and diverse ecology, including both lacustrine and riverine trout from the Drin-Skadar drainage in Albania, a known hotspot of Salmonid (genetic) diversity [3].

First, our results pointed out a single MHC-*DAB* locus and no evidence of individual copy-number variation across the examined taxa, consistently with previous findings based on European riverine *S. trutta* complex populations (e.g., [12,13,14]). However, this was not entirely predictable since duplicated loci are frequent in Salmonids due to incomplete diploidization—specifically, ref. [4] revealed partial duplication of microsatellite loci in a few *S. ohridanus* individuals.

The MHC-*DAB* sequences of Albanian trouts showed intricate genealogies, evident as multiple neighbor-net splits (Figure 1A), even more pronounced when the analysis was extended to all comparable *S. trutta* complex MHC-*DAB* sequences (Figure 1B). This indicates evolution by reticulation (possibly coupled with recombination), that applies well to species complexes where gene flow among taxa is not fully prevented [30]. Despite profound divergence (>4 Mya) and evolution in peculiar ecological conditions (i.e., a deep-water lacustrine environment), the *S. ohridanus* sequences did not have deeper divergence compared to the others (Figure 1), which may imply trans-species polymorphism. Although (adaptive) introgression may lead to patterns consistent with trans-species polymorphisms [8], the latter is more likely because of the prolonged reproductive isolation of *S. ohridanus* [1,4].

Typical MHC polymorphism patterns emerged for *S. letnica* and *S. trutta* complex sequences. Besides a lack of in-frame stop codons, we found high nucleotide sequence diversity, mostly leading to amino acid replacement, especially at ABSs (Table 2), and outstanding MHC allelic diversity, as opposed to relatively homogeneous neutral mitochondrial diversity within populations (Table 1). Both findings indicate prominent roles for (historical) positive selection and balancing selection in increasing and maintaining MHC diversity over evolutionary time [8,9]. The trout PSSs inferred in this study strongly matched human-derived ABSs (*p* = 0.0033; Fisher’s exact test) and represented a subset (except for amino acid positions 7 and 68) of 28 PSSs previously identified using 139 brown trout MHC-*DAB* sequences and similar methods [12], confirming cross-taxa conservation of MHC protein architecture.

Even though our sampling was only partially representative of the *Salmo* diversity around the examined area (increasing the sample sizes would provide a more accurate characterization of MHC variation), overall we found remarkable MHC allelic diversity in our samples compared to natural European populations, either native or hatchery-introgressed. In fact, as a rough measure of MHC variability, the mean number of MHC-*DAB* alleles per individual ranged between 0.86 (*S. letnica*) and 1.20 (the *S. trutta* complex population from Skadar) in our samples, while it measured 0.14–0.41 in *S. trutta* complex populations from three Austrian basins [14], 0.05–0.65 in populations from multiple sampling sites in the Sella River drainage (northern Spain) [13], and 0.27–0.93 in six unrelated populations in central Italy [12]. Consistent with previous studies targeting putatively neutral variation (mitochondrial DNA and microsatellites) in Albanian trout populations [4,5], we found multiple indications of strong population/taxon distinctiveness at MHC-*DAB*: (1) high global/pairwise Fst and D values (Table 3); (2) the occurrence of just a single allele shared between the Cem and Skadar populations (i.e., Satr-DAB*120; Figure S2A); and (3) only 4 of 34 detected alleles matching 139 known *S. trutta* complex alleles that were mostly detected across European populations. The relative contributions of evolutionary processes (e.g., local selection, genetic drift, and gene flow) to the observed MHC-based structuring remain uncertain due to a lack of explicit comparisons with neutral genome-wide variation (e.g., microsatellites or SNPs) [9]. It is worth mentioning that high allele diversity did not yield functional richness (only four inferred supertypes), and no evident functional distinctiveness emerged from the patterns of supertype distribution across taxa or ecological forms (i.e., riverine vs. lacustrine trout) (Figure S2B and Table 1).

Interestingly, we found relatively reduced MHC-*DAB* sequence and allelic diversity in *S. ohridanus* (Table 1 and Table 2) coupled with relaxed positive selection (no significant dN > dS values in any partition; Table 2), apparently suggesting diversity erosion due to genetic drift (e.g., [14]). On the other hand, the retention of evolutionarily neutral mitochondrial and nuclear diversity ([4] and this study) appears partly inconsistent with such a hypothesis, which would entail pathogen-driven directional selection reducing MHC (but not evolutionarily neutral) variation [9]. This result seems to corroborate the occurrence of a single functional supertype (Figure 1) and adaptation of *S. ohridanus* to a peculiar deep-water environment. However, any hypothesis should be accepted with caution because of the small sample size. Further investigations and increased sampling may clarify this intriguing outcome.

## 5. Conclusions

Our findings provide a basis for further investigations aimed at analyzing ecological (i.e., local parasites and lacustrine vs. riverine environments) and evolutionary drivers yielding the high MHC diversity and structuring harbored within the Western Balkan Salmonid hotspot. We reiterate the importance of preserving, through conservation programs, adaptive variation and the evolutionary potential of wild populations to ensure their long-term viability.

## Figures and Tables

**Table 1:** The genetic variation at the MHC-*DAB* gene across trout taxa and populations from Albania: sample size (N); number of alleles (A); allelic richness (Ar); observed (Ho) and expected (He) heterozygosity with corresponding *p*-values (values < 0.05 are given in bold) from the Hardy–Weinberg equilibrium test (*p*); and frequencies of MHC supertypes. The frequencies of Control Region haplotypes and their mitochondrial lineages are also provided. Haplotypes marked with an asterisk matched with multiple GenBank accessions (Table S1).

			Control Region		MHC-*DAB*					
Taxon	Location	N	Lineage	Haplotypes (GenBank)	A	Ar	Ho	He	*p*	Supertypes
*S. ohridanus*	Ohrid Lake	3	*ohridanus*	Ohr-3 (AY926568) × 1; Ohr-6 (AY926559) × 1; Ohr-1 * (AY926564) × 1	3	2.55	0.67	0.60	0.861	ST4 (100%)
*S. letnica*	Ohrid Lake	22	Adriatic (AD)-*letnica*	Let12 (AY926570) × 17; Let13 (AY926573) × 2; Let15 (AY926572) × 1; Let16 (DQ381568) × 2	19	5.11	0.82	0.94	0.154	ST1 (25.0%); ST2 (27.3%); ST3 (38.6%); ST4 (9.1%)
*S. trutta* complex	Cem River	6	Adriatic (AD)	AD-cs11 * (AY836340) × 6	7	4.15	0.33	0.88	**0.022**	ST1 (16.7%); ST2 (16.7%); ST3 (16.6%); ST4 (50.0%)
*S. trutta* complex	Skadar Lake	5	Adriatic (AD)	AD-cs11 * (AY836340) × 4	6	4.07	1.00	0.89	0.246	ST1 (70.0%); ST3 (30.0%)

**Table 2:** The sequence polymorphism of MHC-*DAB* for trout taxa and three alignment partitions: predicted ABSs based on human MHC, non-ABSs, and all sites. The average nucleotide (Kimura two-parameter model) and amino acid (Poisson-corrected) distances are shown with standard errors (SEs), and the Z-statistics for the dN > dS test are shown with the corresponding *p*-values (significant ones are presented in bold).

Taxon	Alleles	Partition	Nucleotide (±SE)	Amino Acid (±SE)	dN (±SE)	dS (±SE)	Z	*p*-Value
*S. ohridanus*	3	all sites	0.019 (0.007)	0.041 (0.018)	0.021 (0.010)	0.012 (0.012)	0.763	0.224
		ABSs	0.071 (0.026)	0.157 (0.071)	0.077 (0.034)	0.050 (0.053)	0.498	0.310
		non-ABSs	0.000 (0.000)	0.000 (0.000)	0.000 (0.000)	0.000 (0.000)	0.000	1.000
*S. letnica*	19	all sites	0.097 (0.013)	0.200 (0.033)	0.110 (0.018)	0.041 (0.014)	3.211	**0.001**
		ABSs	0.223 (0.041)	0.492 (0.097)	0.271 (0.056)	0.073 (0.036)	3.197	**0.001**
		non-ABSs	0.056 (0.010)	0.111 (0.028)	0.057 (0.014)	0.031 (0.014)	1.358	0.088
*S. trutta* complex	12	all sites	0.094 (0.014)	0.190 (0.031)	0.104 (0.017)	0.044 (0.019)	2.347	**0.010**
		ABSs	0.214 (0.040)	0.457 (0.096)	0.261 (0.058)	0.065 (0.047)	2.542	**0.006**
		non-ABSs	0.055 (0.011)	0.109 (0.028)	0.053 (0.013)	0.037 (0.021)	0.684	0.248
overall	*34*	all sites	*0.101 (0.014)*	*0.206 (0.034)*	*0.114 (0.019)*	*0.046 (0.017)*	*2.289*	***0.003***
		*ABSs*	*0.229 (0.041)*	*0.493 (0.099)*	*0.276 (0.053)*	*0.079 (0.044)*	*2.928*	***0.002***
		non-ABSs	*0.060 (0.011)*	*0.119 (0.028)*	*0.061 (0.015)*	*0.035 (0.017)*	*1.134*	*0.129*

**Table 3:** Measures of genetic differentiation between population/taxon pairs based on Fst and Jost’s D are given above and below the diagonal, respectively. Estimates that were statistically significant (*p* < 0.05) after the Bonferroni correction are shown in bold.

	*S. ohridanus*	*S. letnica*	*S. trutta* Complex (Cem)	*S. trutta* Complex (Skadar)
*S. ohridanus*	-	**0.17**	0.21	0.21
*S. letnica*	**1.00**	-	**0.07**	**0.08**
*S. trutta* complex (Cem)	1.00	**1.00**	-	0.11
*S. trutta* complex (Skadar)	1.00	**1.00**	0.91	-

## Data Availability

Individual genotypes for MHC-*DAB* and haplotypes for the mitochondrial CR are provided in Table S1. Thirty novel MHC-*DAB* sequences were deposited in GenBank with accessions PQ729919–PQ729948.

## Supplementary Materials

The following supporting information can be downloaded at: https://www.mdpi.com/article/10.3390/biology13121060/s1, Figure S1: Amino acid alignment of 34 MHC-*DAB* alleles; Figure S2: Frequency and distribution of MHC-*DAB* alleles and supertypes across sampling sites; Table S1: individual MHC-*DAB* genotypes and Control Region haplotype sequences.
