# Supplementary material for: Unveiling MHC-DAB Polymorphism Within the Western Balkan Salmonid Hotspot: Preliminary Outcomes from Native Trouts of Ohrid Lake and the Drin-Skadar Drainage (Albania)
Source: Biology (Basel). 2024 Dec 18;13(12):1060. doi: 10.3390/biology13121060 (PMC11673393; doi:10.3390/biology13121060)
Supplement: Supplementary file 1 [file biology-13-01060-s001.zip › biology-3350510-supplementary.pdf]

Supplementary information

Figure S1

Amino acid alignment (85 positions) of 34 MHC-DAB alleles retrieved from overall 36 Albanian samples of *S. ohridanus*, *S. letnica* and *S. trutta* complex– For each allele, the corresponding supertype (ST) is given; non-novel alleles are indicated with GenBank accession in brackets. The "+" signs mark predicted ABS according to human MHC and PSS inferred by each of four performed tests (p: P-value; PP: posterior probability) -- pink-shaded positions highlight 10 PSS detected by at least two tests. The grey-shaded position identifies the recombinant breakpoint detected by the GARD method.

	MHC-DAB allele	ST	1	2	3	4	5	6	7	8	9	10	11	12	13	14	15	16	17	18	19	20	21	22	23	24	25	26	27	28	29	30	31	32	33	34	35	36	37	38	39	40	41	42	43	44	45	46	47	48	49	50	51	52	53	54	55	56	57	58	59	60	61	62	63	64	65	66	67	68	69	70	71	72	73	74	75	76	77	78	79	80	81	82	83	84	85
S.ohridanus	Saoh-DAB*001	4	D	G	Y	F	E	Q	S	V	V	E	C	R	Y	S	S	K	D	L	Q	G	A	E	L	I	D	S	Y	V	F	N	Q	A	E	Y	I	R	F	N	S	T	V	G	K	Y	V	G	Y	T	E	H	G	V	K	N	A	E	A	W	N	K	G	P	E	-	L	A	R	E	L	G	E	L	E	R	V	C	K	H	N	A	A	I	E	Y	S
	Saoh-DAB*002	4	.	.	.	.	.	.	.	.	.	.	.	.	.	.	.	.	.	.	.	.	.	.	.	.	.	.	.	.	.	.	.	.	.	.	.	.	.	.	.	.	.	.	.	.	.	.	.	.	.	.	.	.	.	.	.	.	.	.	.	.	.	.	.	-	.	.	.	.	.	.	.	.	.	.	F	.	.	.	.	.	.	L	H	.	.
	Saoh-DAB*003	4	.	.	.	.	.	.	R	.	.	.	.	.	.	.	.	.	.	.	.	.	.	.	.	.	.	.	.	.	.	.	.	.	.	.	.	.	.	.	.	.	.	.	.	.	.	.	.	.	.	.	.	.	.	.	.	.	.	L	.	.	.	.	.	-	.	.	.	.	.	.	.	.	.	.	F	.	.	.	.	.	.	.	.	.	.
S. letnica	Sale-DAB*001	3	.	.	.	.	.	.	R	.	S	.	.	.	.	.	.	.	.	.	.	.	.	.	F	.	.	.	.	Y	.	.	.	V	.	H	.	.	.	.	.	.	.	.	.	.	.	.	.	.	.	.	.	.	Y	.	.	.	.	.	.	S	D	A	A	F	.	.	Q	.	R	.	.	.	.	.	.	.	.	Q	H	.	P	.	Y	.	.
	Sale-DAB*002	2	.	.	.	.	Y	.	V	.	R	Q	.	.	.	.	.	.	.	.	.	.	I	.	Y	.	.	.	.	.	.	.	K	.	.	H	V	.	.	.	.	.	.	.	.	.	.	.	.	.	.	.	.	.	.	.	.	.	.	.	.	.	.	.	.	-	.	.	Q	.	.	.	Q	.	.	.	Y	.	.	.	.	.	P	.	Y	.	.
	Sale-DAB*003	4	.	.	.	.	H	H	M	M	R	Q	.	.	.	.	.	.	.	.	.	D	.	.	.	.	T	.	.	.	.	.	K	.	.	.	.	.	.	.	.	.	.	.	.	.	.	.	.	.	.	.	.	.	.	.	.	.	.	.	.	S	D	A	A	F	.	.	Q	.	R	.	.	.	.	.	F	.	.	P	.	.	D	L	H	.	N
	Sale-DAB*004 & *012	1	.	.	.	.	.	.	V	.	R	Q	.	.	F	.	.	.	.	.	.	.	I	.	F	.	.	.	.	.	.	.	K	V	.	D	.	.	.	.	.	.	.	.	.	.	.	.	.	.	.	.	.	.	.	.	.	.	.	.	.	.	.	.	.	-	.	.	G	.	.	.	.	.	.	.	.	.	.	P	.	.	.	N	H	.	.
	Sale-DAB*005	1	.	.	.	.	.	.	V	.	R	Q	.	.	F	.	.	.	.	.	.	.	I	.	.	.	.	.	.	.	.	.	K	V	.	D	.	.	.	.	.	.	.	.	.	.	.	.	.	.	.	.	.	.	.	.	.	.	.	.	.	S	D	A	G	I	.	.	Q	M	.	.	.	.	.	.	Y	.	.	P	T	.	.	.	D	.	.
	Sale-DAB*006	3	.	.	.	.	H	.	I	.	S	.	.	.	.	.	.	.	.	.	.	.	I	.	F	.	.	.	.	.	.	.	K	.	.	.	.	.	.	.	.	.	.	.	.	.	.	.	.	.	.	Y	.	.	Y	.	.	.	.	.	.	.	.	.	.	-	.	.	Q	.	.	.	.	.	.	.	.	.	.	.	.	.	P	.	Y	.	.
	Sale-DAB*007	3	.	.	.	.	Y	.	R	.	A	.	.	.	F	.	.	.	.	.	.	.	I	.	F	.	.	.	.	.	.	.	K	.	.	.	.	.	.	.	.	.	.	.	.	.	.	.	.	.	.	Y	.	.	Y	.	.	.	.	.	.	.	.	.	.	-	.	.	Q	.	.	.	.	.	.	.	.	.	.	.	.	.	.	.	D	.	.
	Sale-DAB*008	2	.	.	.	.	.	.	V	.	R	Q	.	.	F	.	.	.	.	.	.	.	I	.	F	.	.	.	.	.	.	.	K	V	.	D	.	.	.	.	.	.	.	.	.	.	.	.	.	.	.	.	.	.	.	.	.	.	.	.	.	.	.	.	.	-	.	.	.	A	.	.	.	.	.	.	Y	.	.	.	.	.	P	.	Y	.	.
	Sale-DAB*009 & *013	2	.	.	.	.	.	.	V	.	R	Q	.	.	.	.	.	.	.	.	.	.	I	.	F	.	.	.	.	.	.	.	K	.	.	H	V	.	.	.	.	.	.	.	.	.	.	.	.	.	.	.	.	.	.	.	.	.	.	.	.	.	.	.	.	-	.	.	.	A	.	.	.	.	.	.	Y	.	.	.	.	.	P	.	Y	.	.
	Sale-DAB*010	3	.	.	.	.	H	.	I	.	S	.	.	.	.	.	.	.	.	.	.	.	I	.	F	.	.	.	.	.	.	.	K	.	.	.	.	.	.	.	.	.	.	.	.	.	.	.	.	.	.	Y	.	.	Y	.	.	.	.	.	.	.	.	.	.	-	.	.	Q	.	.	.	.	.	.	.	.	.	.	.	.	.	.	.	D	.	.
	Sale-DAB*011	2	.	.	.	.	H	.	V	.	K	Q	.	.	F	.	.	.	.	.	.	.	I	.	.	.	.	.	.	.	.	.	.	.	.	.	.	.	.	.	.	.	.	.	.	F	.	.	.	.	.	.	.	.	.	.	.	.	.	.	.	S	D	A	G	I	.	.	Q	.	R	.	.	.	.	.	.	.	.	Q	H	.	P	.	Y	.	.
	Sale-DAB*014	1	.	.	.	.	Y	.	V	.	R	Q	.	.	.	.	.	.	.	.	.	.	I	.	Y	.	.	.	.	.	.	.	K	.	.	H	V	.	.	.	.	.	.	.	.	.	.	.	.	.	.	.	.	.	.	.	.	.	.	.	.	.	.	.	.	-	.	.	Q	.	.	.	Q	.	.	.	Y	.	.	P	.	.	D	L	H	.	.
	Sale-DAB*015	2	.	.	.	.	Y	H	M	M	R	Q	.	.	.	.	.	.	.	.	.	.	I	.	.	.	T	.	.	.	.	.	K	.	.	.	.	.	.	.	.	.	.	.	.	F	.	.	.	.	.	.	.	.	.	.	.	.	.	.	.	.	.	.	.	-	.	.	Q	.	.	.	.	.	.	.	Y	.	.	.	.	.	P	.	Y	.	.
	Sale-DAB*016	2	.	.	.	.	Q	.	R	.	T	.	.	.	.	.	.	.	.	.	.	.	I	.	.	.	.	.	.	.	.	.	.	.	.	.	.	.	.	.	.	.	.	.	.	F	.	.	.	.	.	.	.	.	.	.	.	.	.	.	.	S	D	A	A	F	.	.	Q	M	.	.	.	.	.	.	Y	.	.	.	.	.	P	.	Y	.	.
	Satr-DAB*051 (DQ491053)	3	.	.	.	.	.	H	.	.	.	.	.	.	.	.	.	.	.	.	.	.	I	.	M	.	.	.	.	Y	.	.	K	V	.	D	V	.	.	.	.	.	.	.	.	.	.	.	.	.	.	Q	.	.	Y	Y	.	.	.	.	.	.	.	.	.	-	.	.	G	.	.	.	.	.	.	.	F	.	.	G	S	.	.	.	Y	.	.
	Satr-DAB*0902 (KT003667)	1	.	.	.	.	H	.	V	.	K	Q	.	.	F	.	.	.	.	.	.	.	I	.	.	.	.	.	.	.	.	.	.	.	.	.	.	.	.	.	.	.	.	.	.	.	.	.	.	.	.	.	.	.	.	.	.	.	.	.	.	.	.	.	.	-	.	.	Q	.	.	.	.	.	.	.	.	.	.	P	.	.	.	.	D	.	.
	Satr-DAB*1401a (KT003658)	3	.	.	.	.	N	.	R	.	T	.	.	.	.	.	.	.	.	.	.	.	I	.	Y	.	.	.	.	.	.	.	.	V	.	N	.	.	.	.	.	.	.	.	.	.	.	.	.	.	.	L	.	.	.	.	.	.	.	.	.	.	.	.	.	-	.	.	Q	M	.	.	Q	.	.	.	Y	.	.	P	.	.	D	L	R	.	.
S. trutta complex	Satr-DAB*004 (DQ257390)	1	.	.	.	.	Y	.	R	.	A	.	.	.	F	.	.	.	.	.	.	.	I	.	.	.	.	.	.	.	.	.	.	.	.	.	.	.	.	.	.	.	.	.	.	.	.	.	.	.	.	.	.	.	Y	.	.	.	.	.	.	S	D	A	G	I	.	.	Q	.	R	.	.	.	.	.	.	.	.	.	.	.	D	L	H	.	.
	Satr-DAB*119 & *126	1	.	.	.	.	.	.	V	.	R	Q	.	.	F	.	.	.	.	.	.	.	I	.	.	.	.	.	.	.	.	.	K	V	.	D	.	.	.	.	.	.	.	.	.	.	.	.	.	.	.	.	.	.	.	.	.	.	.	.	.	S	D	A	G	I	.	.	Q	M	.	.	Q	.	.	.	Y	.	.	P	.	.	.	.	D	.	.
	Satr-DAB*120	3	.	.	.	.	Y	.	R	.	A	.	.	.	F	.	.	.	.	.	.	.	I	.	.	.	.	.	.	.	.	.	.	.	.	.	.	.	.	.	.	.	.	.	.	.	.	.	.	.	.	.	.	.	Y	.	.	.	.	.	.	S	D	A	G	I	.	.	Q	.	.	.	.	.	.	.	.	.	.	P	.	.	.	.	D	.	.
	Satr-DAB*121	1	.	.	.	.	H	H	M	M	R	Q	.	.	.	.	.	.	.	.	.	.	I	.	.	.	T	.	.	.	.	.	K	.	.	.	V	.	.	.	.	.	.	.	.	.	.	.	.	.	.	.	.	.	.	.	.	.	.	.	.	S	D	A	A	I	.	.	Q	.	R	.	.	.	.	.	Y	.	.	P	T	.	.	.	D	.	.
	Satr-DAB*122	4	.	.	.	.	H	H	M	M	K	Q	.	.	.	.	.	.	.	.	.	D	.	.	.	.	T	.	.	.	.	.	K	V	.	D	.	.	.	.	.	.	.	.	.	F	.	.	.	.	.	.	.	.	.	.	.	.	.	.	.	S	D	A	G	I	.	.	Q	.	.	.	.	.	.	.	Y	.	.	P	.	.	.	.	D	.	.
	Satr-DAB*123	2	.	.	.	.	H	H	M	M	R	Q	.	.	.	.	.	.	.	.	.	.	I	.	.	.	T	.	.	.	.	.	K	.	.	.	.	.	.	.	.	.	.	.	.	F	.	.	.	.	.	.	.	.	.	.	.	.	.	.	.	.	.	.	.	-	.	.	.	.	.	.	.	.	.	.	.	.	.	.	.	.	P	.	Y	.	.
	Satr-DAB*124	1	.	.	.	.	H	H	M	M	R	Q	.	.	.	.	.	.	.	.	.	.	I	.	.	.	T	.	.	.	.	.	K	.	.	.	V	.	.	.	.	.	.	.	.	.	.	.	.	.	.	.	.	.	.	.	.	.	.	.	.	S	D	A	A	I	.	.	Q	.	R	.	.	.	.	.	.	.	.	P	.	.	.	.	Y	.	.
	Satr-DAB*125	1	.	.	.	.	H	H	M	M	R	Q	.	.	.	.	.	.	.	.	.	.	I	.	.	.	T	.	.	.	.	.	K	.	.	.	V	.	.	.	.	.	.	.	.	.	.	.	.	.	.	.	.	.	.	.	.	.	.	.	.	S	D	A	A	I	.	.	Q	.	R	.	.	.	.	.	Y	.	.	P	.	.	.	.	Y	.	.
	Satr-DAB*127	4	.	.	.	.	H	.	R	.	S	.	.	.	.	.	.	.	.	.	.	.	.	.	Y	.	N	.	.	.	.	.	K	V	.	N	.	.	.	.	.	.	.	.	.	.	.	.	.	.	.	L	.	.	.	.	.	.	.	.	.	.	.	.	.	-	.	.	Q	M	.	.	.	.	.	.	F	.	.	.	.	.	P	.	Y	.	.
	Satr-DAB*128	3	.	.	.	.	Q	.	R	.	T	.	.	.	.	.	.	.	.	.	.	.	I	.	.	.	.	.	.	.	.	.	.	.	.	.	.	.	.	.	.	.	.	.	.	F	.	.	.	.	.	.	.	.	Y	.	.	.	.	.	.	S	D	A	G	I	.	.	Q	M	.	.	.	.	.	.	.	.	.	P	.	.	.	.	D	.	.
	Satr-DAB*129	1	.	.	.	.	.	.	V	.	R	Q	.	.	F	.	.	.	.	.	.	.	I	.	.	.	.	.	.	.	.	.	K	V	.	D	.	.	.	.	.	.	.	.	.	.	.	.	.	.	.	.	.	.	Y	.	.	.	.	.	.	S	D	A	A	F	.	.	Q	M	.	.	.	.	.	.	Y	.	.	F	.	.	D	L	R	.	.
	**MEME** (p<0.05)								+		+												+				+			+																									+							+							+	+							+			+			+		+		
	**FEL** (p<0.05)										+				+								+																																																							+			+			+		+		
	**SLAC** (p<0.05)										+																																																																				+			+			+				
	**FUBAR** (PP>0.95)						+		+		+				+								+		+									+		+																			+										+					+							+			+			+		+		
	Human ABS						+		+		+																+		+		+					+	+									+									+				+	+				+			+		+	+			+				+			+	+			+	+		+

Figure S2

Frequency and spatial distribution of 34 MHC-DAB alleles (A) and 4 functional supertypes (B) across wild populations of *S. ohridanus*, *S. letnica* and *S. trutta* complex (Skadar Lake and Cem River) from the Drin-Skadar drainage in Albania.

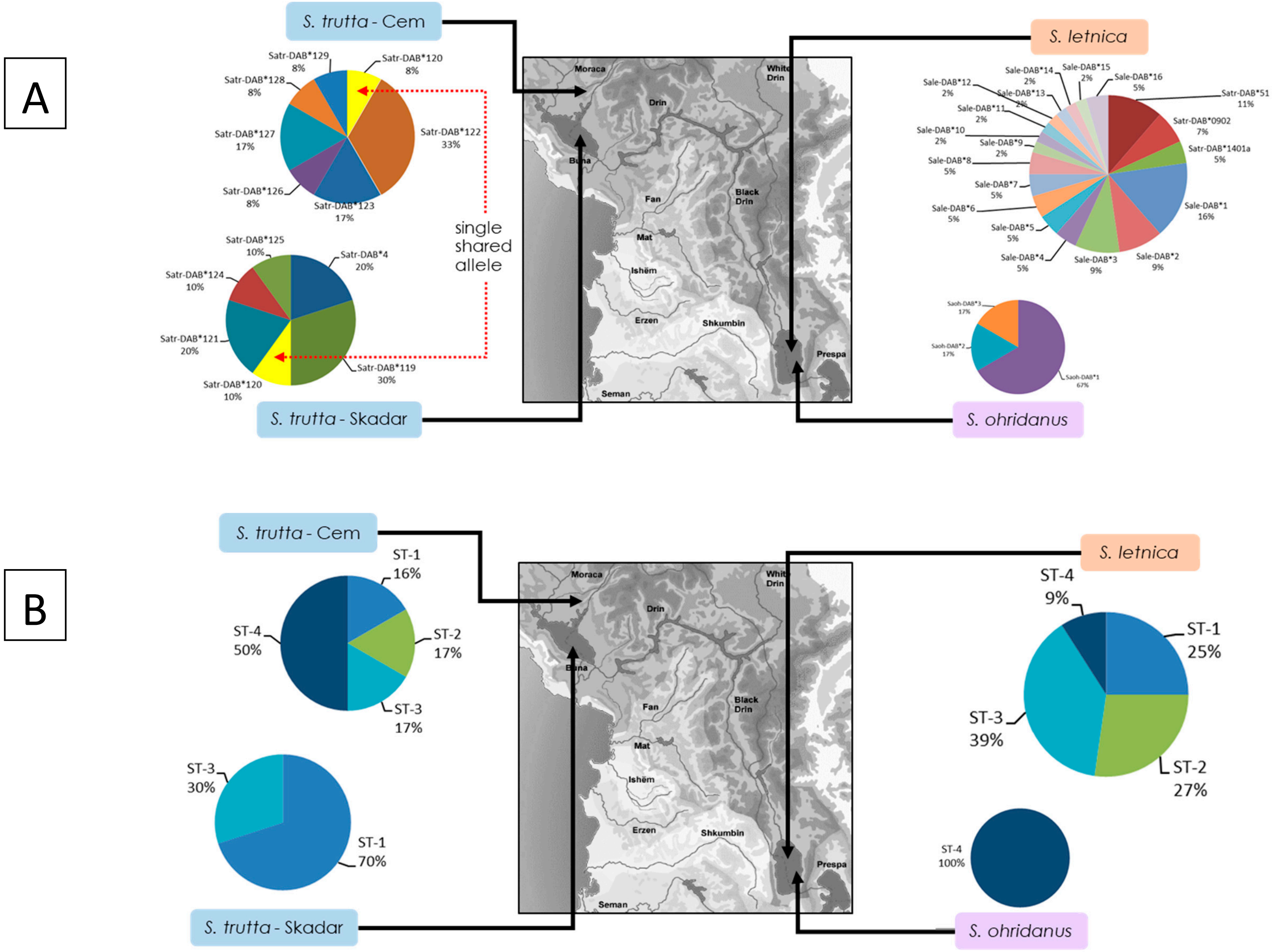

Table S1

Details on MHC-DAB genotype and Control Region haplotype for 3 *Salmo ohridanus*, 22 *S. letnica* and 11 *S. trutta* complex specimens.

Taxon	Specimen code	Location	MHC-DAB genotype	Control Region haplotype (GenBank #)	Contro Region sequence
Salmo letnica	Sale-OHR_001	Ohrid Lake	Satr-DAB*051 / Sale-DAB*001	Let12 (AY926570)	CAGCTATGTACAATAACAACTGTTGTACCTTGCTAACCCAATGTTATACTACATCTATGTATAATATTACATATTATGTATTTAC<br>CCATATATATAATATAGCATGTGAGTAGTACATCATATGTATTATCAACATTAGTGAATTTAACCCCTCATACATCAGCACTAA<br>CTCAAGGTTTACATAAAGCAAAACACGTGATAATAACCAACTAAGTTGTCTTAACCCGATTAATTGTTATATCAATAAAACTCC<br>ACCTAACACGGGCTCCGTCTTTACCCACCAACTTTCAGCATCAGTCCTGCTTAATGTAGTAAGAACCGACCAACGATATATCA<br>GTAGGCATACTCTTATTGATGGTCAGGGACAGATATCGTATTAGGTCGCATCTCGTGAACTATTCCTGGCATTTGGTTCCTAT<br>ATCAAGGGCTATCCTTAAGAAACCACCCCCTGAAAGCCGAATGTAAAGCATCTGGTTAATGGTGTCAATCTTATTGCCCGTTA<br>CCCACCAAGCCGGGCGTTCTCTTATATGCATAGGGTTCCCTTTTTTTTTTTTT
Salmo letnica	Sale-OHR_002	Ohrid Lake	Sale-DAB*004 / Sale-DAB*005	Let16 (DQ381568)	CAGCTATGTACAATAACAACTGTTGTACCTTGCTAACCCGATGTTATACTACATCTATGTATAATATTACATATTATGTATTTAC<br>CCATATATATAATATAGCATGTGAGTAGTACATCATATGTATTATCAACATTAGTGAATTTAACCCCTCATACATCAGCACTAA<br>CTCAAGGTTTACATAAAGCAAAACACGTGATAATAACCAACTAAGTTGTCTTAACCCGATTAATTGTTATATCAATAAAACTCC<br>ACCTAACACGGGCTCCGTCTTTACCCACCAACTTTCAGCATCAGTCCTGCTTAATGTAGTAAGAACCGACCAACGATATATCA<br>GTAGGCATACTCTTATTGATGGTCAGGGACAGATATCGTATTAGGTCGCATCTCGTGAACTATTCCTGGCATTTGGTTCCTAT<br>ATCAAGGGCTATCCTTAAGAAACCACCCCCTGAAAGCCGAATGTAAAGCATCTGGTTAATGGTGTCAATCTTATTGCCCGTTA<br>CCCACCAAGCCGGGCGTTCTCTTATATGCATAGGGTTCCCTTTTTTTTTTTTT
Salmo letnica	Sale-OHR_003	Ohrid Lake	Sale-DAB*007 / Sale-DAB*012	Let12 (AY926570)	CAGCTATGTACAATAACAACTGTTGTACCTTGCTAACCCAATGTTATACTACATCTATGTATAATATTACATATTATGTATTTAC<br>CCATATATATAATATAGCATGTGAGTAGTACATCATATGTATTATCAACATTAGTGAATTTAACCCCTCATACATCAGCACTAA<br>CTCAAGGTTTACATAAAGCAAAACACGTGATAATAACCAACTAAGTTGTCTTAACCCGATTAATTGTTATATCAATAAAACTCC<br>ACCTAACACGGGCTCCGTCTTTACCCACCAACTTTCAGCATCAGTCCTGCTTAATGTAGTAAGAACCGACCAACGATATATCA<br>GTAGGCATACTCTTATTGATGGTCAGGGACAGATATCGTATTAGGTCGCATCTCGTGAACTATTCCTGGCATTTGGTTCCTAT<br>ATCAAGGGCTATCCTTAAGAAACCACCCCCTGAAAGCCGAATGTAAAGCATCTGGTTAATGGTGTCAATCTTATTGCCCGTTA<br>CCCACCAAGCCGGGCGTTCTCTTATATGCATAGGGTTCCCTTTTTTTTTTTTT
Salmo letnica	Sale-OHR_004	Ohrid Lake	Sale-DAB*010 / Sale-DAB*013	Let12 (AY926570)	CAGCTATGTACAATAACAACTGTTGTACCTTGCTAACCCAATGTTATACTACATCTATGTATAATATTACATATTATGTATTTAC<br>CCATATATATAATATAGCATGTGAGTAGTACATCATATGTATTATCAACATTAGTGAATTTAACCCCTCATACATCAGCACTAA<br>CTCAAGGTTTACATAAAGCAAAACACGTGATAATAACCAACTAAGTTGTCTTAACCCGATTAATTGTTATATCAATAAAACTCC<br>ACCTAACACGGGCTCCGTCTTTACCCACCAACTTTCAGCATCAGTCCTGCTTAATGTAGTAAGAACCGACCAACGATATATCA<br>GTAGGCATACTCTTATTGATGGTCAGGGACAGATATCGTATTAGGTCGCATCTCGTGAACTATTCCTGGCATTTGGTTCCTAT<br>ATCAAGGGCTATCCTTAAGAAACCACCCCCTGAAAGCCGAATGTAAAGCATCTGGTTAATGGTGTCAATCTTATTGCCCGTTA<br>CCCACCAAGCCGGGCGTTCTCTTATATGCATAGGGTTCCCTTTTTTTTTTTTT
Salmo letnica	Sale-OHR_005	Ohrid Lake	Sale-DAB*006 / Sale-DAB*009	Let12 (AY926570)	CAGCTATGTACAATAACAACTGTTGTACCTTGCTAACCCAATGTTATACTACATCTATGTATAATATTACATATTATGTATTTAC<br>CCATATATATAATATAGCATGTGAGTAGTACATCATATGTATTATCAACATTAGTGAATTTAACCCCTCATACATCAGCACTAA<br>CTCAAGGTTTACATAAAGCAAAACACGTGATAATAACCAACTAAGTTGTCTTAACCCGATTAATTGTTATATCAATAAAACTCC<br>ACCTAACACGGGCTCCGTCTTTACCCACCAACTTTCAGCATCAGTCCTGCTTAATGTAGTAAGAACCGACCAACGATATATCA<br>GTAGGCATACTCTTATTGATGGTCAGGGACAGATATCGTATTAGGTCGCATCTCGTGAACTATTCCTGGCATTTGGTTCCTAT<br>ATCAAGGGCTATCCTTAAGAAACCACCCCCTGAAAGCCGAATGTAAAGCATCTGGTTAATGGTGTCAATCTTATTGCCCGTTA<br>CCCACCAAGCCGGGCGTTCTCTTATATGCATAGGGTTCCCTTTTTTTTTTTTT
Salmo letnica	Sale-OHR_006	Ohrid Lake	Sale-DAB*016 / Sale-DAB*016	Let12 (AY926570)	CAGCTATGTACAATAACAACTGTTGTACCTTGCTAACCCAATGTTATACTACATCTATGTATAATATTACATATTATGTATTTAC<br>CCATATATATAATATAGCATGTGAGTAGTACATCATATGTATTATCAACATTAGTGAATTTAACCCCTCATACATCAGCACTAA<br>CTCAAGGTTTACATAAAGCAAAACACGTGATAATAACCAACTAAGTTGTCTTAACCCGATTAATTGTTATATCAATAAAACTCC<br>ACCTAACACGGGCTCCGTCTTTACCCACCAACTTTCAGCATCAGTCCTGCTTAATGTAGTAAGAACCGACCAACGATATATCA<br>GTAGGCATACTCTTATTGATGGTCAGGGACAGATATCGTATTAGGTCGCATCTCGTGAACTATTCCTGGCATTTGGTTCCTAT<br>ATCAAGGGCTATCCTTAAGAAACCACCCCCTGAAAGCCGAATGTAAAGCATCTGGTTAATGGTGTCAATCTTATTGCCCGTTA<br>CCCACCAAGCCGGGCGTTCTCTTATATGCATAGGGTTCCCTTTTTTTTTTTTT
Salmo letnica	Sale-OHR_007	Ohrid Lake	Satr-DAB*051 / Satr-DAB*051	Let16 (DQ381568)	CAGCTATGTACAATAACAACTGTTGTACCTTGCTAACCCGATGTTATACTACATCTATGTATAATATTACATATTATGTATTTAC<br>CCATATATATAATATAGCATGTGAGTAGTACATCATATGTATTATCAACATTAGTGAATTTAACCCCTCATACATCAGCACTAA<br>CTCAAGGTTTACATAAAGCAAAACACGTGATAATAACCAACTAAGTTGTCTTAACCCGATTAATTGTTATATCAATAAAACTCC<br>ACCTAACACGGGCTCCGTCTTTACCCACCAACTTTCAGCATCAGTCCTGCTTAATGTAGTAAGAACCGACCAACGATATATCA

					GTAGGCATACTCTTATTGATGGTCAGGGACAGATATCGTATTAGGTCGCATCTCGTGAACTATTCCTGGCATTTGGTTCCTAT<br>ATCAAGGGCTATCCTTAAGAAACCACCCCCTGAAAGCCGAATGTAAAGCATCTGGTTAATGGTGTCAATCTTATTGCCCGTTA<br>CCCACCAAGCCGGGCGTTCTCTTATATGCATAGGGTTCCCTTTTTTTTTTTTT
Salmo letnica	Sale-OHR_008	Ohrid Lake	Sale-DAB*002 / Sale-DAB*006	Let12 (AY926570)	CAGCTATGTACAATAACAACTGTTGTACCTTGCTAACCCAATGTTATACTACATCTATGTATAATATTACATATTATGTATTTAC<br>CCATATATATAATATAGCATGTGAGTAGTACATCATATGTATTATCAACATTAGTGAATTTAACCCCTCATACATCAGCACTAA<br>CTCAAGGTTTACATAAAGCAAAACACGTGATAATAACCAACTAAGTTGTCTTAACCCGATTAATTGTTATATCAATAAAACTCC<br>ACCTAACACGGGCTCCGTCTTTACCCACCAACTTTCAGCATCAGTCCTGCTTAATGTAGTAAGAACCGACCAACGATATATCA<br>GTAGGCATACTCTTATTGATGGTCAGGGACAGATATCGTATTAGGTCGCATCTCGTGAACTATTCCTGGCATTTGGTTCCTAT<br>ATCAAGGGCTATCCTTAAGAAACCACCCCCTGAAAGCCGAATGTAAAGCATCTGGTTAATGGTGTCAATCTTATTGCCCGTTA<br>CCCACCAAGCCGGGCGTTCTCTTATATGCATAGGGTTCCCTTTTTTTTTTTTT
Salmo letnica	Sale-OHR_009	Ohrid Lake	Satr-DAB*0902 / Satr-DAB*1401a	Let12 (AY926570)	CAGCTATGTACAATAACAACTGTTGTACCTTGCTAACCCAATGTTATACTACATCTATGTATAATATTACATATTATGTATTTAC<br>CCATATATATAATATAGCATGTGAGTAGTACATCATATGTATTATCAACATTAGTGAATTTAACCCCTCATACATCAGCACTAA<br>CTCAAGGTTTACATAAAGCAAAACACGTGATAATAACCAACTAAGTTGTCTTAACCCGATTAATTGTTATATCAATAAAACTCC<br>ACCTAACACGGGCTCCGTCTTTACCCACCAACTTTCAGCATCAGTCCTGCTTAATGTAGTAAGAACCGACCAACGATATATCA<br>GTAGGCATACTCTTATTGATGGTCAGGGACAGATATCGTATTAGGTCGCATCTCGTGAACTATTCCTGGCATTTGGTTCCTAT<br>ATCAAGGGCTATCCTTAAGAAACCACCCCCTGAAAGCCGAATGTAAAGCATCTGGTTAATGGTGTCAATCTTATTGCCCGTTA<br>CCCACCAAGCCGGGCGTTCTCTTATATGCATAGGGTTCCCTTTTTTTTTTTTT
Salmo letnica	Sale-OHR_010	Ohrid Lake	Satr-DAB*1401a / Sale-DAB*004	Let12 (AY926570)	CAGCTATGTACAATAACAACTGTTGTACCTTGCTAACCCAATGTTATACTACATCTATGTATAATATTACATATTATGTATTTAC<br>CCATATATATAATATAGCATGTGAGTAGTACATCATATGTATTATCAACATTAGTGAATTTAACCCCTCATACATCAGCACTAA<br>CTCAAGGTTTACATAAAGCAAAACACGTGATAATAACCAACTAAGTTGTCTTAACCCGATTAATTGTTATATCAATAAAACTCC<br>ACCTAACACGGGCTCCGTCTTTACCCACCAACTTTCAGCATCAGTCCTGCTTAATGTAGTAAGAACCGACCAACGATATATCA<br>GTAGGCATACTCTTATTGATGGTCAGGGACAGATATCGTATTAGGTCGCATCTCGTGAACTATTCCTGGCATTTGGTTCCTAT<br>ATCAAGGGCTATCCTTAAGAAACCACCCCCTGAAAGCCGAATGTAAAGCATCTGGTTAATGGTGTCAATCTTATTGCCCGTTA<br>CCCACCAAGCCGGGCGTTCTCTTATATGCATAGGGTTCCCTTTTTTTTTTTTT
Salmo letnica	Sale-OHR_011	Ohrid Lake	Sale-DAB*001 / Sale-DAB*008	Let12 (AY926570)	CAGCTATGTACAATAACAACTGTTGTACCTTGCTAACCCAATGTTATACTACATCTATGTATAATATTACATATTATGTATTTAC<br>CCATATATATAATATAGCATGTGAGTAGTACATCATATGTATTATCAACATTAGTGAATTTAACCCCTCATACATCAGCACTAA<br>CTCAAGGTTTACATAAAGCAAAACACGTGATAATAACCAACTAAGTTGTCTTAACCCGATTAATTGTTATATCAATAAAACTCC<br>ACCTAACACGGGCTCCGTCTTTACCCACCAACTTTCAGCATCAGTCCTGCTTAATGTAGTAAGAACCGACCAACGATATATCA<br>GTAGGCATACTCTTATTGATGGTCAGGGACAGATATCGTATTAGGTCGCATCTCGTGAACTATTCCTGGCATTTGGTTCCTAT<br>ATCAAGGGCTATCCTTAAGAAACCACCCCCTGAAAGCCGAATGTAAAGCATCTGGTTAATGGTGTCAATCTTATTGCCCGTTA<br>CCCACCAAGCCGGGCGTTCTCTTATATGCATAGGGTTCCCTTTTTTTTTTTTT
Salmo letnica	Sale-OHR_012	Ohrid Lake	Sale-DAB*007 / Sale-DAB*008	Let12 (AY926570)	CAGCTATGTACAATAACAACTGTTGTACCTTGCTAACCCAATGTTATACTACATCTATGTATAATATTACATATTATGTATTTAC<br>CCATATATATAATATAGCATGTGAGTAGTACATCATATGTATTATCAACATTAGTGAATTTAACCCCTCATACATCAGCACTAA<br>CTCAAGGTTTACATAAAGCAAAACACGTGATAATAACCAACTAAGTTGTCTTAACCCGATTAATTGTTATATCAATAAAACTCC<br>ACCTAACACGGGCTCCGTCTTTACCCACCAACTTTCAGCATCAGTCCTGCTTAATGTAGTAAGAACCGACCAACGATATATCA<br>GTAGGCATACTCTTATTGATGGTCAGGGACAGATATCGTATTAGGTCGCATCTCGTGAACTATTCCTGGCATTTGGTTCCTAT<br>ATCAAGGGCTATCCTTAAGAAACCACCCCCTGAAAGCCGAATGTAAAGCATCTGGTTAATGGTGTCAATCTTATTGCCCGTTA<br>CCCACCAAGCCGGGCGTTCTCTTATATGCATAGGGTTCCCTTTTTTTTTTTTT
Salmo letnica	Sale-OHR_014	Ohrid Lake	Sale-DAB*001 / Sale-DAB*001	Let13 (AY926573)	CAGCTATGTACAATAACAACTGTTGTACCTTGCTAACCCAATGTTATACTACATCTATGTATAATATTACATATTATGTATTTAC<br>CCATATATATAATATAGCATGTGAGTAGTACATCATATGTATTATCAACATTAGTGAATTTAACCCCTCATACATCAGCACTAA<br>CTCAAGGTTTACATAAAGCAAAACACGTGATAATAACCAACTAAGTTGTCTTAACCCGATTAATTGTTATATCAATAAAACTCC<br>ACCTAACACGGGCTCCGTCTTTACCCACCAACTTTCAGCATCAGTCCTGCTTAATGTAGTAAGAACCGACCAACGATATATCA<br>GTAGGCATACTCTTATTGATGGTCAGGGACAGATATCGTATTAGGTCGCATCTCGTGAACTATTCCTGGCATTTGGTTCCTAT<br>ATCAAGGGCTATCCTTAAGAAACCACCCCCTGAAAGCCGAATGTAAAGCATCTGGTTAATGGTGTCAATCTTATTGCCCGTTA<br>CCCACCAAGCCGGGCGTTCTCTTATATGCATAGGGTTCTCTTTTTTTTTTTTT
Salmo letnica	Sale-OHR_015	Ohrid Lake	Satr-DAB*0902 / Sale-DAB*005	Let12 (AY926570)	CAGCTATGTACAATAACAACTGTTGTACCTTGCTAACCCAATGTTATACTACATCTATGTATAATATTACATATTATGTATTTAC<br>CCATATATATAATATAGCATGTGAGTAGTACATCATATGTATTATCAACATTAGTGAATTTAACCCCTCATACATCAGCACTAA<br>CTCAAGGTTTACATAAAGCAAAACACGTGATAATAACCAACTAAGTTGTCTTAACCCGATTAATTGTTATATCAATAAAACTCC<br>ACCTAACACGGGCTCCGTCTTTACCCACCAACTTTCAGCATCAGTCCTGCTTAATGTAGTAAGAACCGACCAACGATATATCA<br>GTAGGCATACTCTTATTGATGGTCAGGGACAGATATCGTATTAGGTCGCATCTCGTGAACTATTCCTGGCATTTGGTTCCTAT<br>ATCAAGGGCTATCCTTAAGAAACCACCCCCTGAAAGCCGAATGTAAAGCATCTGGTTAATGGTGTCAATCTTATTGCCCGTTA<br>CCCACCAAGCCGGGCGTTCTCTTATATGCATAGGGTTCCCTTTTTTTTTTTTT
Salmo letnica	Sale-OHR_016	Ohrid Lake	Satr-DAB*051 / Sale-DAB*015	Let15 (AY926572)	CAGCTATGTACAATAACAACTGTTGTACCTTGCTAACCCAATGTTATGCTACATCTATGTATAATATTACATATTATGTATTTAC<br>CCATATATATAATATAGCATGTGAGTAGTACATCATATGTATTATCAACATTAATGAATTTAACCCCTCATACATCAGCACTAA<br>CTCAAGGTTTACATAAAGCAAAACACGTGATAATAACCAACTAAGTTGTCTTAACCCGATTAATTGTTATATCAATAAAACTCC<br>ACCTAACACGGGCTCCGTCTTTACCCACCAACTTTCAGCATCAGTCCTGCTTAATGTAGTAAGAACCGACCAACGATATATCA<br>GTAGGCATACTCTTATTGATGGTCAGGGACAGATATCGTATTAGGTCGCATCTCGTGAACTATTCCTGGCATTTGGTTCCTAT

					ATCAAGGGCTATCCTTAAGAAACCACCCCCTGAAAGCCGAATGTAAGGCATCTGGTTAATGGTGTCAATCTTATTGCCCGTTA<br>CCCACCAAGCCGGGCGTTCTCTTATATGCATAGGGTTCTCTTTTTTTTTTTTT
Salmo letnica	Sale-OHR_019	Ohrid Lake	Sale-DAB*001 / Sale-DAB*002	Let13 (AY926573)	CAGCTATGTACAATAACAACTGTTGTACCTTGCTAACCCAATGTTATACTACATCTATGTATAATATTACATATTATGTATTTAC<br>CCATATATATAATATAGCATGTGAGTAGTACATCATATGTATTATCAACATTAGTGAATTTAACCCCTCATACATCAGCACTAA<br>CTCAAGGTTTACATAAAGCAAAACACGTGATAATAACCAACTAAGTTGTCTTAACCCGATTAATTGTTATATCAATAAAACTCC<br>ACCTAACACGGGCTCCGTCTTTACCCACCAACTTTCAGCATCAGTCCTGCTTAATGTAGTAAGAACCGACCAACGATATATCA<br>GTAGGCATACTCTTATTGATGGTCAGGGACAGATATCGTATTAGGTCGCATCTCGTGAACTATTCCTGGCATTTGGTTCCTAT<br>ATCAAGGGCTATCCTTAAGAAACCACCCCCTGAAAGCCGAATGTAAAGCATCTGGTTAATGGTGTCAATCTTATTGCCCGTTA<br>CCCACCAAGCCGGGCGTTCTCTTATATGCATAGGGTTCTCTTTTTTTTTTTTT
Salmo letnica	Sale-OHR_020	Ohrid Lake	Sale-DAB*003 / Sale-DAB*003	Let12 (AY926570)	CAGCTATGTACAATAACAACTGTTGTACCTTGCTAACCCAATGTTATACTACATCTATGTATAATATTACATATTATGTATTTAC<br>CCATATATATAATATAGCATGTGAGTAGTACATCATATGTATTATCAACATTAGTGAATTTAACCCCTCATACATCAGCACTAA<br>CTCAAGGTTTACATAAAGCAAAACACGTGATAATAACCAACTAAGTTGTCTTAACCCGATTAATTGTTATATCAATAAAACTCC<br>ACCTAACACGGGCTCCGTCTTTACCCACCAACTTTCAGCATCAGTCCTGCTTAATGTAGTAAGAACCGACCAACGATATATCA<br>GTAGGCATACTCTTATTGATGGTCAGGGACAGATATCGTATTAGGTCGCATCTCGTGAACTATTCCTGGCATTTGGTTCCTAT<br>ATCAAGGGCTATCCTTAAGAAACCACCCCCTGAAAGCCGAATGTAAAGCATCTGGTTAATGGTGTCAATCTTATTGCCCGTTA<br>CCCACCAAGCCGGGCGTTCTCTTATATGCATAGGGTTCCCTTTTTTTTTTTTT
Salmo letnica	Sale-OHR_021	Ohrid Lake	Satr-DAB*051 / Sale-DAB*014	Let12 (AY926570)	CAGCTATGTACAATAACAACTGTTGTACCTTGCTAACCCAATGTTATACTACATCTATGTATAATATTACATATTATGTATTTAC<br>CCATATATATAATATAGCATGTGAGTAGTACATCATATGTATTATCAACATTAGTGAATTTAACCCCTCATACATCAGCACTAA<br>CTCAAGGTTTACATAAAGCAAAACACGTGATAATAACCAACTAAGTTGTCTTAACCCGATTAATTGTTATATCAATAAAACTCC<br>ACCTAACACGGGCTCCGTCTTTACCCACCAACTTTCAGCATCAGTCCTGCTTAATGTAGTAAGAACCGACCAACGATATATCA<br>GTAGGCATACTCTTATTGATGGTCAGGGACAGATATCGTATTAGGTCGCATCTCGTGAACTATTCCTGGCATTTGGTTCCTAT<br>ATCAAGGGCTATCCTTAAGAAACCACCCCCTGAAAGCCGAATGTAAAGCATCTGGTTAATGGTGTCAATCTTATTGCCCGTTA<br>CCCACCAAGCCGGGCGTTCTCTTATATGCATAGGGTTCCCTTTTTTTTTTTTT
Salmo letnica	Sale-OHR_022	Ohrid Lake	Sale-DAB*001 / Sale-DAB*003	Let12 (AY926570)	CAGCTATGTACAATAACAACTGTTGTACCTTGCTAACCCAATGTTATACTACATCTATGTATAATATTACATATTATGTATTTAC<br>CCATATATATAATATAGCATGTGAGTAGTACATCATATGTATTATCAACATTAGTGAATTTAACCCCTCATACATCAGCACTAA<br>CTCAAGGTTTACATAAAGCAAAACACGTGATAATAACCAACTAAGTTGTCTTAACCCGATTAATTGTTATATCAATAAAACTCC<br>ACCTAACACGGGCTCCGTCTTTACCCACCAACTTTCAGCATCAGTCCTGCTTAATGTAGTAAGAACCGACCAACGATATATCA<br>GTAGGCATACTCTTATTGATGGTCAGGGACAGATATCGTATTAGGTCGCATCTCGTGAACTATTCCTGGCATTTGGTTCCTAT<br>ATCAAGGGCTATCCTTAAGAAACCACCCCCTGAAAGCCGAATGTAAAGCATCTGGTTAATGGTGTCAATCTTATTGCCCGTTA<br>CCCACCAAGCCGGGCGTTCTCTTATATGCATAGGGTTCCCTTTTTTTTTTTTT
Salmo letnica	Sale-OHR_023	Ohrid Lake	Sale-DAB*001 / Sale-DAB*011	Let12 (AY926570)	CAGCTATGTACAATAACAACTGTTGTACCTTGCTAACCCAATGTTATACTACATCTATGTATAATATTACATATTATGTATTTAC<br>CCATATATATAATATAGCATGTGAGTAGTACATCATATGTATTATCAACATTAGTGAATTTAACCCCTCATACATCAGCACTAA<br>CTCAAGGTTTACATAAAGCAAAACACGTGATAATAACCAACTAAGTTGTCTTAACCCGATTAATTGTTATATCAATAAAACTCC<br>ACCTAACACGGGCTCCGTCTTTACCCACCAACTTTCAGCATCAGTCCTGCTTAATGTAGTAAGAACCGACCAACGATATATCA<br>GTAGGCATACTCTTATTGATGGTCAGGGACAGATATCGTATTAGGTCGCATCTCGTGAACTATTCCTGGCATTTGGTTCCTAT<br>ATCAAGGGCTATCCTTAAGAAACCACCCCCTGAAAGCCGAATGTAAAGCATCTGGTTAATGGTGTCAATCTTATTGCCCGTTA<br>CCCACCAAGCCGGGCGTTCTCTTATATGCATAGGGTTCCCTTTTTTTTTTTTT
Salmo letnica	Sale-OHR_024	Ohrid Lake	Satr-DAB*0902 / Sale-DAB*002	Let12 (AY926570)	CAGCTATGTACAATAACAACTGTTGTACCTTGCTAACCCAATGTTATACTACATCTATGTATAATATTACATATTATGTATTTAC<br>CCATATATATAATATAGCATGTGAGTAGTACATCATATGTATTATCAACATTAGTGAATTTAACCCCTCATACATCAGCACTAA<br>CTCAAGGTTTACATAAAGCAAAACACGTGATAATAACCAACTAAGTTGTCTTAACCCGATTAATTGTTATATCAATAAAACTCC<br>ACCTAACACGGGCTCCGTCTTTACCCACCAACTTTCAGCATCAGTCCTGCTTAATGTAGTAAGAACCGACCAACGATATATCA<br>GTAGGCATACTCTTATTGATGGTCAGGGACAGATATCGTATTAGGTCGCATCTCGTGAACTATTCCTGGCATTTGGTTCCTAT<br>ATCAAGGGCTATCCTTAAGAAACCACCCCCTGAAAGCCGAATGTAAAGCATCTGGTTAATGGTGTCAATCTTATTGCCCGTTA<br>CCCACCAAGCCGGGCGTTCTCTTATATGCATAGGGTTCCCTTTTTTTTTTTTT
Salmo letnica	Sale-OHR_025	Ohrid Lake	Sale-DAB*002 / Sale-DAB*003	Let12 (AY926570)	CAGCTATGTACAATAACAACTGTTGTACCTTGCTAACCCAATGTTATACTACATCTATGTATAATATTACATATTATGTATTTAC<br>CCATATATATAATATAGCATGTGAGTAGTACATCATATGTATTATCAACATTAGTGAATTTAACCCCTCATACATCAGCACTAA<br>CTCAAGGTTTACATAAAGCAAAACACGTGATAATAACCAACTAAGTTGTCTTAACCCGATTAATTGTTATATCAATAAAACTCC<br>ACCTAACACGGGCTCCGTCTTTACCCACCAACTTTCAGCATCAGTCCTGCTTAATGTAGTAAGAACCGACCAACGATATATCA<br>GTAGGCATACTCTTATTGATGGTCAGGGACAGATATCGTATTAGGTCGCATCTCGTGAACTATTCCTGGCATTTGGTTCCTAT<br>ATCAAGGGCTATCCTTAAGAAACCACCCCCTGAAAGCCGAATGTAAAGCATCTGGTTAATGGTGTCAATCTTATTGCCCGTTA<br>CCCACCAAGCCGGGCGTTCTCTTATATGCATAGGGTTCCCTTTTTTTTTTTTT
Salmo ohridanus	Saoh-OHR_001	Ohrid Lake	Saoh-DAB*001 / Saoh-DAB*001	Ohr3 (AY926568)	CAGCTATGTACAATAACAATTGTTGTACCTTGCTAACCCAATGTTATACTACATCTATGTATAATATTACATATTATGTATTTAC<br>CCATATATATAATATAGCATGTGAGTAGTACATCATATGTATTATCAACATTAATGAATTTAACCCCTCATACATCAGCACTAA<br>CTCAAGGTTTACATAAAGCAAAACACGTGATAATAACCAACTAAGTTGTTTTAACCCGATTAATTGTTATATCAATAAACCTCC<br>AGCTAACACGGGCTCCGTCTTTACCCACCAACTTTCAGCATCAGTCCTGCTTAATGTAGTAAGAACCGACCAACGATTTATCA<br>GTAGGCATACTCTTATTGATGGTCAGGGACAGATATCGTATTGAGTCGCATCTCGTGAACTATTCCTGGCATTTGGTTCCTAT

					ATCAAGGGCTATCCTTAAGAAACCACCCCCTGAAAGCCGAATGTAAGGCATCTGGTTAATGGTGTCAATCTTATTGCCCGTTA<br>CCCACCAAGCCGGGCGTTCTTTTATATGCATAGGGTTCCCTTTTTTTTTTTTT
Salmo ohridanus	Saoh-OHR_002	Ohrid Lake	Saoh-DAB*001 / Saoh-DAB*002	Ohr6 (AY926559)	CAGCTATGTACAATAACAATTGTTGTACCTTGCTAACCCAATGTTATACTACATCTATGTATAATATTACATATTATGTATTTAC<br>CCATATATATAATATAGCATGTGAGTAGTACATCATATGTATTATCAACATTAGTGAATTTAACCCCTCATACATCAGCACTAA<br>CTCAAGGTTTACATAAAGCAAAACACGTGATAATAACCAACTAAGTTGTTTTAACCCGATTAATTGTTATATCAATAAACCTCC<br>AGCTAACACGGGCTCCGTCTTTACCCACCAACTTTCAGCATCAGTCCTGCTTAATGTAGTAAGAACCGACCAACGATTTATCA<br>GTAGGCATACTCTTATTGATGGTCAGGGACAGATATCGTATTAAGTCGCATCTAGTGAACTATTCCTGGCATTTGGTTCCTAT<br>ATCAAGGGCTATCCTTAAGAAACCACCCCCTGAAAGCCGAATGTAAGGCATCTGGTTAATGGTGTCAATCTTATTGCCCGTTA<br>CCCACCAAGCCGGGCGTTCTTTTATATGCATAGGGTTCCCTTTTTTTTTTTTT
Salmo ohridanus	Saoh-OHR_003	Ohrid Lake	Saoh-DAB*001 / Saoh-DAB*003	Ohr1 (AY926564)<br>Ohr2 (AY926560)<br>Ohr8 (AY926567)<br>Ohr9 (AY926565)<br>Ohr10 (AY926562)<br>Ohr11 (AY926566)	CAGCTATGTACAATAACAATTGTTGTACCTTGCTAACCCAATGTTATACTACATCTATGTATAATATTACATATTATGTATTTAC<br>CCATATATATAATATAGCATGTGAGTAGTACATCATATGTATTATCAACATTAGTGAATTTAACCCCTCATACATCAGCACTAA<br>CTCAAGGTTTACATAAAGCAAAACACGTGATAATAACCAACTAAGTTGTTTTAACCCGATTAATTGTTATATCAATAAACCTCC<br>AGCTAACACGGGCTCCGTCTTTACCCACCAACTTTCAGCATCAGTCCTGCTTAATGTAGTAAGAACCGACCAACGATTTATCA<br>GTAGGCATACTCTTATTGATGGTCAGGGACAGATATCGTATTAAGTCGCATCTCGTGAACTATTCCTGGCATTTGGTTCCTAT<br>ATCAAGGGCTATCCTTAAGAAACCACCCCCTGAAAGCCGAATGTAAGGCATCTGGTTAATGGTGTCAATCTTATTGCCCGTTA<br>CCCACCAAGCCGGGCGTTCTTTTATATGCATAGGGTTCCCTTTTTTTTTTTTT
Salmo trutta complex	Satr-CEM_007	Cem River	Satr-DAB*122 / Satr-DAB*122	AD-cs11 (AY836340)<br>AD-Tyrrh1 (KX450258)	CAGCTATGTACAATAACAACTGTTGTACCTTGCTAACCCAATGTTATACTACATCTATGTATAATATTACATACTATGTATTTAC<br>CCATATATATAATATAGCATGTGAGTAGTACATCATATGTATTATCAACATTAGTGAATTTAACCCCTCATACATCAGCACTAA<br>CTCAAGGTTTACATAAAGCAAAACACGTGATAATAACCAACTAAGTTGTCTTAACCCGATTAATTGTTATATCAATAAAACTCC<br>ACCTAACACGGGCTCCGTCTTTACCCACCAACTTTCAGCATCAGTCCTGCTTAATGTAGTAAGAACCGACCAACGATATATCA<br>GTAGGCATACTCTTATTGATGGTCAGGGACAGATATCGTATTAGGTCGCATCTCGTGAACTATTCCTGGCATTTGGTTCCTAT<br>ATCAAGGGCTATCCTTAAGAAACCACCCCCTGAAAGCCGAATGTAAAGCATCTGGTTAATGGTGTCAATCTTATTGCCCGTTA<br>CCCACCAAGCCGGGCGTTCTTTTATATGCATAGGGTTCCCTTTTTTTTTTTTT
Salmo trutta complex	Satr-CEM_008	Cem River	Satr-DAB*127 / Satr-DAB*127	AD-cs11 (AY836340)<br>AD-Tyrrh1 (KX450258	CAGCTATGTACAATAACAACTGTTGTACCTTGCTAACCCAATGTTATACTACATCTATGTATAATATTACATACTATGTATTTAC<br>CCATATATATAATATAGCATGTGAGTAGTACATCATATGTATTATCAACATTAGTGAATTTAACCCCTCATACATCAGCACTAA<br>CTCAAGGTTTACATAAAGCAAAACACGTGATAATAACCAACTAAGTTGTCTTAACCCGATTAATTGTTATATCAATAAAACTCC<br>ACCTAACACGGGCTCCGTCTTTACCCACCAACTTTCAGCATCAGTCCTGCTTAATGTAGTAAGAACCGACCAACGATATATCA<br>GTAGGCATACTCTTATTGATGGTCAGGGACAGATATCGTATTAGGTCGCATCTCGTGAACTATTCCTGGCATTTGGTTCCTAT<br>ATCAAGGGCTATCCTTAAGAAACCACCCCCTGAAAGCCGAATGTAAAGCATCTGGTTAATGGTGTCAATCTTATTGCCCGTTA<br>CCCACCAAGCCGGGCGTTCTTTTATATGCATAGGGTTCCCTTTTTTTTTTTTT
Salmo trutta complex	Satr-CEM_009	Cem River	Satr-DAB*123 / Satr-DAB*123	AD-cs11 (AY836340)<br>AD-Tyrrh1 (KX450258	CAGCTATGTACAATAACAACTGTTGTACCTTGCTAACCCAATGTTATACTACATCTATGTATAATATTACATACTATGTATTTAC<br>CCATATATATAATATAGCATGTGAGTAGTACATCATATGTATTATCAACATTAGTGAATTTAACCCCTCATACATCAGCACTAA<br>CTCAAGGTTTACATAAAGCAAAACACGTGATAATAACCAACTAAGTTGTCTTAACCCGATTAATTGTTATATCAATAAAACTCC<br>ACCTAACACGGGCTCCGTCTTTACCCACCAACTTTCAGCATCAGTCCTGCTTAATGTAGTAAGAACCGACCAACGATATATCA<br>GTAGGCATACTCTTATTGATGGTCAGGGACAGATATCGTATTAGGTCGCATCTCGTGAACTATTCCTGGCATTTGGTTCCTAT<br>ATCAAGGGCTATCCTTAAGAAACCACCCCCTGAAAGCCGAATGTAAAGCATCTGGTTAATGGTGTCAATCTTATTGCCCGTTA<br>CCCACCAAGCCGGGCGTTCTTTTATATGCATAGGGTTCCCTTTTTTTTTTTTT
Salmo trutta complex	Satr-CEM_010	Cem River	Satr-DAB*120 / Satr-DAB*129	AD-cs11 (AY836340)<br>AD-Tyrrh1 (KX450258	CAGCTATGTACAATAACAACTGTTGTACCTTGCTAACCCAATGTTATACTACATCTATGTATAATATTACATACTATGTATTTAC<br>CCATATATATAATATAGCATGTGAGTAGTACATCATATGTATTATCAACATTAGTGAATTTAACCCCTCATACATCAGCACTAA<br>CTCAAGGTTTACATAAAGCAAAACACGTGATAATAACCAACTAAGTTGTCTTAACCCGATTAATTGTTATATCAATAAAACTCC<br>ACCTAACACGGGCTCCGTCTTTACCCACCAACTTTCAGCATCAGTCCTGCTTAATGTAGTAAGAACCGACCAACGATATATCA<br>GTAGGCATACTCTTATTGATGGTCAGGGACAGATATCGTATTAGGTCGCATCTCGTGAACTATTCCTGGCATTTGGTTCCTAT<br>ATCAAGGGCTATCCTTAAGAAACCACCCCCTGAAAGCCGAATGTAAAGCATCTGGTTAATGGTGTCAATCTTATTGCCCGTTA<br>CCCACCAAGCCGGGCGTTCTTTTATATGCATAGGGTTCCCTTTTTTTTTTTTT
Salmo trutta complex	Satr-CEM_011	Cem River	Satr-DAB*122 / Satr-DAB*122	AD-cs11 (AY836340)<br>AD-Tyrrh1 (KX450258	CAGCTATGTACAATAACAACTGTTGTACCTTGCTAACCCAATGTTATACTACATCTATGTATAATATTACATACTATGTATTTAC<br>CCATATATATAATATAGCATGTGAGTAGTACATCATATGTATTATCAACATTAGTGAATTTAACCCCTCATACATCAGCACTAA<br>CTCAAGGTTTACATAAAGCAAAACACGTGATAATAACCAACTAAGTTGTCTTAACCCGATTAATTGTTATATCAATAAAACTCC<br>ACCTAACACGGGCTCCGTCTTTACCCACCAACTTTCAGCATCAGTCCTGCTTAATGTAGTAAGAACCGACCAACGATATATCA<br>GTAGGCATACTCTTATTGATGGTCAGGGACAGATATCGTATTAGGTCGCATCTCGTGAACTATTCCTGGCATTTGGTTCCTAT<br>ATCAAGGGCTATCCTTAAGAAACCACCCCCTGAAAGCCGAATGTAAAGCATCTGGTTAATGGTGTCAATCTTATTGCCCGTTA<br>CCCACCAAGCCGGGCGTTCTTTTATATGCATAGGGTTCCCTTTTTTTTTTTTT
Salmo trutta complex	Satr-CEM_012	Cem River	Satr-DAB*126 / Satr-DAB*128	AD-cs11 (AY836340)<br>AD-Tyrrh1 (KX450258	CAGCTATGTACAATAACAACTGTTGTACCTTGCTAACCCAATGTTATACTACATCTATGTATAATATTACATACTATGTATTTAC<br>CCATATATATAATATAGCATGTGAGTAGTACATCATATGTATTATCAACATTAGTGAATTTAACCCCTCATACATCAGCACTAA<br>CTCAAGGTTTACATAAAGCAAAACACGTGATAATAACCAACTAAGTTGTCTTAACCCGATTAATTGTTATATCAATAAAACTCC<br>ACCTAACACGGGCTCCGTCTTTACCCACCAACTTTCAGCATCAGTCCTGCTTAATGTAGTAAGAACCGACCAACGATATATCA<br>GTAGGCATACTCTTATTGATGGTCAGGGACAGATATCGTATTAGGTCGCATCTCGTGAACTATTCCTGGCATTTGGTTCCTAT

					ATCAAGGGCTATCCTTAAGAAACCACCCCCTGAAAGCCGAATGTAAAGCATCTGGTTAATGGTGTCAATCTTATTGCCCGTTA<br>CCCACCAAGCCGGGCGTTCTTTTATATGCATAGGGTTCCCTTTTTTTTTTTTT
Salmo trutta complex	Satr-SCU_001	Skadar Lake	Satr-DAB*119 / Satr-DAB*121	-	-
Salmo trutta complex	Satr-SCU_002	Skadar Lake	Satr-DAB*121 / Satr-DAB*125	AD-cs11 (AY836340)<br>AD-Tyrrh1 (KX450258	CAGCTATGTACAATAACAACTGTTGTACCTTGCTAACCCAATGTTATACTACATCTATGTATAATATTACATACTATGTATTTAC<br>CCATATATATAATATAGCATGTGAGTAGTACATCATATGTATTATCAACATTAGTGAATTTAACCCCTCATACATCAGCACTAA<br>CTCAAGGTTTACATAAAGCAAAACACGTGATAATAACCAACTAAGTTGTCTTAACCCGATTAATTGTTATATCAATAAAACTCC<br>ACCTAACACGGGCTCCGTCTTTACCCACCAACTTTCAGCATCAGTCCTGCTTAATGTAGTAAGAACCGACCAACGATATATCA<br>GTAGGCATACTCTTATTGATGGTCAGGGACAGATATCGTATTAGGTCGCATCTCGTGAACTATTCCTGGCATTTGGTTCCTAT<br>ATCAAGGGCTATCCTTAAGAAACCACCCCCTGAAAGCCGAATGTAAAGCATCTGGTTAATGGTGTCAATCTTATTGCCCGTTA<br>CCCACCAAGCCGGGCGTTCTTTTATATGCATAGGGTTCCCTTTTTTTTTTTTT
Salmo trutta complex	Satr-SCU_003	Skadar Lake	Satr-DAB*124 / Satr-DAB*120	AD-cs11 (AY836340)<br>AD-Tyrrh1 (KX450258	CAGCTATGTACAATAACAACTGTTGTACCTTGCTAACCCAATGTTATACTACATCTATGTATAATATTACATACTATGTATTTAC<br>CCATATATATAATATAGCATGTGAGTAGTACATCATATGTATTATCAACATTAGTGAATTTAACCCCTCATACATCAGCACTAA<br>CTCAAGGTTTACATAAAGCAAAACACGTGATAATAACCAACTAAGTTGTCTTAACCCGATTAATTGTTATATCAATAAAACTCC<br>ACCTAACACGGGCTCCGTCTTTACCCACCAACTTTCAGCATCAGTCCTGCTTAATGTAGTAAGAACCGACCAACGATATATCA<br>GTAGGCATACTCTTATTGATGGTCAGGGACAGATATCGTATTAGGTCGCATCTCGTGAACTATTCCTGGCATTTGGTTCCTAT<br>ATCAAGGGCTATCCTTAAGAAACCACCCCCTGAAAGCCGAATGTAAAGCATCTGGTTAATGGTGTCAATCTTATTGCCCGTTA<br>CCCACCAAGCCGGGCGTTCTTTTATATGCATAGGGTTCCCTTTTTTTTTTTTT
Salmo trutta complex	Satr-SCU_004	Skadar Lake	Satr-DAB*004 / Satr-DAB*119	AD-cs11 (AY836340)<br>AD-Tyrrh1 (KX450258	CAGCTATGTACAATAACAACTGTTGTACCTTGCTAACCCAATGTTATACTACATCTATGTATAATATTACATACTATGTATTTAC<br>CCATATATATAATATAGCATGTGAGTAGTACATCATATGTATTATCAACATTAGTGAATTTAACCCCTCATACATCAGCACTAA<br>CTCAAGGTTTACATAAAGCAAAACACGTGATAATAACCAACTAAGTTGTCTTAACCCGATTAATTGTTATATCAATAAAACTCC<br>ACCTAACACGGGCTCCGTCTTTACCCACCAACTTTCAGCATCAGTCCTGCTTAATGTAGTAAGAACCGACCAACGATATATCA<br>GTAGGCATACTCTTATTGATGGTCAGGGACAGATATCGTATTAGGTCGCATCTCGTGAACTATTCCTGGCATTTGGTTCCTAT<br>ATCAAGGGCTATCCTTAAGAAACCACCCCCTGAAAGCCGAATGTAAAGCATCTGGTTAATGGTGTCAATCTTATTGCCCGTTA<br>CCCACCAAGCCGGGCGTTCTTTTATATGCATAGGGTTCCCTTTTTTTTTTTTT
Salmo trutta complex	Satr-SCU_005	Skadar Lake	Satr-DAB*004 / Satr-DAB*119	AD-cs11 (AY836340)<br>AD-Tyrrh1 (KX450258	CAGCTATGTACAATAACAACTGTTGTACCTTGCTAACCCAATGTTATACTACATCTATGTATAATATTACATACTATGTATTTAC<br>CCATATATATAATATAGCATGTGAGTAGTACATCATATGTATTATCAACATTAGTGAATTTAACCCCTCATACATCAGCACTAA<br>CTCAAGGTTTACATAAAGCAAAACACGTGATAATAACCAACTAAGTTGTCTTAACCCGATTAATTGTTATATCAATAAAACTCC<br>ACCTAACACGGGCTCCGTCTTTACCCACCAACTTTCAGCATCAGTCCTGCTTAATGTAGTAAGAACCGACCAACGATATATCA<br>GTAGGCATACTCTTATTGATGGTCAGGGACAGATATCGTATTAGGTCGCATCTCGTGAACTATTCCTGGCATTTGGTTCCTAT<br>ATCAAGGGCTATCCTTAAGAAACCACCCCCTGAAAGCCGAATGTAAAGCATCTGGTTAATGGTGTCAATCTTATTGCCCGTTA<br>CCCACCAAGCCGGGCGTTCTTTTATATGCATAGGGTTCCCTTTTTTTTTTTTT
